# Emerging insights on intestinal dysbiosis during bacterial infections^☆^

**DOI:** 10.1016/j.mib.2013.12.002

**Published:** 2014-02

**Authors:** Tu Anh N Pham, Trevor D Lawley

**Affiliations:** Wellcome Trust Sanger Institute, Wellcome Trust Genome Campus, Hinxton CB10 1SA, United Kingdom

## Abstract

Infection of the gastrointestinal tract is commonly linked to pathological imbalances of the resident microbiota, termed dysbiosis. In recent years, advanced high-throughput genomic approaches have allowed us to examine the microbiota in an unprecedented manner, revealing novel biological insights about infection-associated dysbiosis at the community and individual species levels. A dysbiotic microbiota is typically reduced in taxonomic diversity and metabolic function, and can harbour pathobionts that exacerbate intestinal inflammation or manifest systemic disease. Dysbiosis can also promote pathogen genome evolution, while allowing the pathogens to persist at high density and transmit to new hosts. A deeper understanding of bacterial pathogenicity in the context of the intestinal microbiota should unveil new approaches for developing diagnostics and therapies for enteropathogens.

## Highlights

• Diverse enteric pathogens often induce significant perturbations to the microbiota or thrive during dysbiosis.
• Infection-associated dysbiosis is commonly characterized by decreased diversity and metabolic function.
• The dysbiotic microbiota may act as a pathogenic community to perpetuate host pathology.
• Pathogens can exploit dysbiosis for host colonization, genome evolution, and transmission.
• Bacteriotherapy represents a potential viable strategy to restore intestinal homeostasis.

**Current Opinion in Microbiology** 2014, **17**:67–74

This review comes from a themed issue on **Host–microbe interactions: bacteria**

Edited by **Olivia Steele-Mortimer** and **Agathe Subtil**

For a complete overview see the Issue and the Editorial

Available online 29th December 2013

1369-5274/$ – see front matter, © 2013 The Authors. Published by Elsevier Ltd. All rights reserved.

**http://dx.doi.org/10.1016/j.mib.2013.12.002**

## Introduction

The human intestinal microbiota is composed of 500–1000 diverse species, which together contains approximately 150 times more unique genes than our genome [1]. Often viewed as a “digestive organ”, the microbiota has co-evolved with the host to form a complex mutualistic relationship [2]: the gastrointestinal tract provides a nourishing environment for its microbial community, while the microbiota performs a wide range of essential metabolic, developmental and immune functions. A health-associated microbiota also represents the first line of defence against invading pathogens or resident opportunists, and can facilitate pathogen clearance from the intestinal tract [3].

Over the past decade, the development of high-throughput sequencing technologies and analysis tools has enabled us to study the microbiota at an exceptional depth and resolution. At the same time, there is an increasing recognition that many pathogens such as *Clostridium difficile* and enterococci harbour potent virulence factors in their genomes, yet are commonly associated with asymptomatic carriage. Thus a pathogen's ability to manifest virulence versus commensalism cannot be determined from the genome alone [4^•^], and virulence genes (e.g. those encoding bacterial toxins, antimicrobial resistance, adhesion factors) may be essentially viewed as colonization factors [4^•^,5]. Disease manifestation often depends not only on the dynamic between the pathogens and host immunity, but also on the composition and activity of the cohabiting microbiota. Recent studies monitoring the microbiota in patients or murine models of bacterial infection have indeed revealed new insights about pathogen biology during dysbiosis, including host colonization, disease, adaptation and transmission. Below, we discuss emerging concepts on infection-associated dysbiosis and their implications for host–microbe interactions.

## The intestinal microbiota during homeostasis

Without exposure to antibiotics or enteropathogens, a healthy gastrointestinal tract is home to a dense and diverse microbial community, known as the microbiota. A typical intestinal microbiota is dominated by obligate anaerobes belonging to the phyla Bacteroidetes, Firmicutes and Actinobacteria, and facultative anaerobes of the Proteobacteria phylum [6]. The microbiota assembly and structure vary widely between different individuals and at different anatomical sites along the length of the intestinal tract [7]. Nevertheless, a health-associated microbiome (that is, the collective encoding potential of the microbiota) is believed to be functionally conserved, and contains a shared gene set necessary to perform important biochemical reactions for host physiology [8^•^]. These functions include the degradation of xenobiotic substances, vitamin biosynthesis and fermentation of indigestible polysaccharides into beneficial short-chain fatty acids (SCFA). Colonization by microbes also promotes our immune development, including the generation of IgA-secreting plasma cells or regulatory T cells to establish intestinal homeostasis with the commensal microbiota [9]. Finally, a healthy gut ecosystem is essential for colonization resistance [10,11,12^•^], whereby both the microbial community and the basal immune responses against resident commensals can together prevent access of pathogens.

## The intestinal microbiota during infections

**Figure 1:**
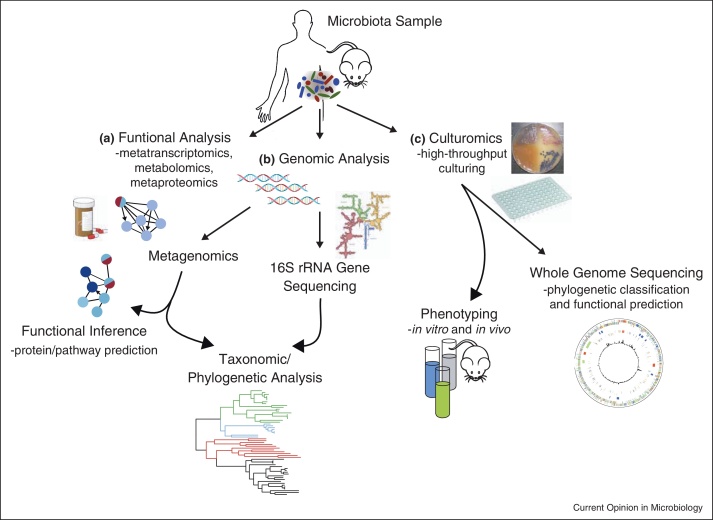
High-throughput genomic techniques commonly applied in microbiota research. **(a)** The functional state of the microbiota can be assessed directly by measuring its transcriptome (i.e. RNA-sequencing or metatranscriptomics), proteome (i.e. metaproteomics) or metabolites (i.e. metabolomics). Such approaches are still in their infancy but hold great promise for developing microbiota-based therapies and assessing human clinical studies. **(b)** Microbiota composition and taxonomy can be determined through directed amplicon sequencing of the 16S rRNA genes or by extracting 16S rRNA gene data from metagenomic datasets. Direct sequencing of the total DNA (i.e. shotgun metagenomics) also allows a measurement of the community function by defining the proteins and pathways (e.g. KEGG, COG, RefSeq pathways) that could potentially be active in the community to infer the overall functional capacity of the community. **(c)** Microbial species from the microbiota may be isolated and cultured by high-throughput techniques, termed “culturomics”, such as the use of barcoded plates with rich non-selective agar or liquid medium. The resulting microbes can then be whole-genome sequenced to examine their genetic traits, or analysed biologically with *in vitro* or *in vivo* assays. A combination of these complementary approaches will expand our understanding of the microbiota during health and disease and may ultimately yield microbiota-based therapeutics and diagnostics.

The importance of the resident microbiota during intestinal infections was highlighted by two seminal papers utilizing murine infection models with the Gram-negative pathogens *Salmonella enterica* serovar Typhimurium (or *S.* Typhimurium [13^••^]) and *Citrobacter rodentium* [14^••^]. In both models, pathogen-induced inflammation either led to or stabilized an imbalanced state of the microbiota community structure and function, termed intestinal dysbiosis. Advanced genomic methods have since been applied to other infection models, including Gram-positive and Gram-negative pathogens, to further define dysbiosis at both the microbial community and single species levels (Box 1 and Figure 1). Below, we summarize some of the emerging concepts from these studies.

### Exploitation of dysbiosis by enteric pathogens

Diverse enteric pathogens often exploit dysbiosis, whether precipitated by antibiotic use or host inflammation, to outcompete resident commensals and gain access to intestinal nutrients and niches. In mice, *Salmonella* Typhimurium and *Clostridium difficile* can both colonize the gut asymptomatically but only overgrow to high density and induce pathology after antibiotic treatment [13^••^,15]. *C. difficile* is also the leading cause of antibiotic-associated diarrhea in humans, whereby disease manifestation predominantly occurs following antibiotic disruption of the microbiota, or in patients with inflammatory bowel disease [16]. When dysbiosis occurs, pathogens can rapidly outcompete commensals due to a greater resistance to host defences (e.g. antimicrobial and phagocyte killing), and better utilization of the gut nutrient environment [12^•^,17]. For example, *Salmonella*'s competitive advantage is partly conferred by the ability to overcome host sequestration of iron [18] and to respire anaerobically using reactive oxygen species derived from the inflamed gut [19]. The metabolic environment during dysbiosis is also high in the SCFAs acetate and formate, which positively regulate the expression of *Salmonella* pathogenicity island-1 [20,21]. In addition, antibiotic use can lead to an increased availability of mucosal carbohydrates that are normally consumed by commensal *Bacteroides*, thus opening up new replicative niches for pathogens such as *Salmonella* and *C. difficile* [22].

### Dysbiosis is characterized by a simplified community structure and function

Characterization of intestinal dysbiosis by different 16S rRNA gene sequencing approaches has consistently shown a reduction in taxonomic diversity and species membership of the microbiota. This observation also holds true across multiple human studies and animal infection models, including *S.* Typhimurium, *C. rodentium*, *C. difficile*, and vancomycin-resistant enterococci (VRE) [14^••^,23–25,26^••^,27^•^,28]. The overall bacterial biomass may decrease in some cases depending on the inflammatory insult [14^••^,29]. In addition, dysbiosis generally leads to a depletion of obligate anaerobic bacteria such as *Bacteroides* and *Ruminococcus* spp., and conversely, a bloom in facultative anaerobes including the family Enterobacteriaceae (e.g. *E. coli*, *Klebsiella* spp., *Proteus* spp.). This shift may partly be due to the ability of Enterobacteriaceae species to respire using reactive nitrogen species — a byproduct of host inflammation, thereby outcompeting other commensals [30]. However, the complex mechanism underlying other population-wide changes during dysbiosis (e.g. the bloom of anaerobic *Prevotella* spp. driven by NLRP6 inflammasome deficiency [31]) remains unclear.

Among the functional consequences of a simplified microbiota is a reduced metabolic capacity, often exemplified by a decline in SCFA production. This outcome may be in part due to a reduction in anaerobic bacteria, including dominant SCFA-producing genera such as *Bacteroides*, *Clostridium*, *Bifidobacterium* and *Roseburia*. SCFAs are physiological byproducts of carbohydrate fermentation by the microbiota, and serve to salvage energy for the host as well as to enhance the mucosal barrier, inhibit intestinal inflammation and oxidative stress [32]. Dysbiosis caused by broad-spectrum antibiotics (e.g. clindamycin, cephalosporins), which can trigger opportunistic infection by *C. difficile* and enterococci, is commonly associated with low intestinal SCFA levels [23,33]. Furthermore, *C. difficile* infection may itself lead to decreased amounts of faecal acetate and butyrate, both in humans and equivalent murine models [25,33]. In the streptomycin-induced model of *S.* Typhimurium infection, butyrate level also decreases in the large intestine, which may promote bacterial invasion by stimulating expression of the *Salmonella* pathogenicity island genes [34]. As such, the microbiota's declining metabolic capacity may further impair host defence to pathogens and promote the stability of a dysbiotic community.

### The dysbiotic microbiota acts as a pathogenic community

In *S*. Typhimurium infection, a microbiota with simplified structure (e.g. in mice treated with clinically relevant doses of antibiotics) or increased Enterobacteriaceae abundance may exacerbate disease outcome [35,36^•^,37]. The pathogenic role of a dysbiotic microbiota is also shown in *C. difficile*-associated diarrhea, in which dysbiosis caused by an epidemic *C. difficile* strain leads to relapsing infection with more severe pathology [25]. Interestingly in some infection models such as *C. difficile* [25] and *C. rodentium* [14^••^,28], microbiota analyses reveal that the inciting pathogens often constitute only a minor fraction of the overall microbial community. Together, these findings suggest that low-abundance pathogens could induce global changes to the microbiota structure and function, in a manner that further destabilizes the intestinal ecosystem. Such enteropathogens may be considered ‘keystone species’ [38^••^], and likely influence the microbial community through a combination of their virulence expression, and of the host inflammatory and metabolic responses.

A dysbiotic microbiota may also be enriched for pathobionts — resident species with virulence potential that are normally kept at low levels. An overgrowth of commensal Enterobacteriaceae (*Klebsiella* spp. and *Proteus* spp.) or *Helicobacter typhlonius* has been shown to occur during intestinal dysbiosis, and can directly trigger colitis in mice [39]. Moreover, the depletion of anaerobic commensals during dysbiosis can lead to intestinal overgrowth of VRE, both in murine infection models or patients undergoing antibiotic therapies [26^••^,40]. This consequently predisposes the host to invasive enterococcal infections with life-threatening sequelae [26^••^]. Similarly, a multidrug-resistant *E. coli* pathobiont can expand in the mouse intestine following antibiotic disruption of the microbiota, causing bacteremia and sepsis [41]. Therefore during dysbiosis, the host may be increasingly susceptible to both pathogens and pathobionts, and the microbiota may be viewed collectively as a pathogenic community.

### An ecosystem for pathogen virulence expression and genome evolution

The microbiota often influences pathogen virulence and fitness upon passage through the gastrointestinal tract. Signaling from commensal bacteria has been shown to upregulate the virulence genes of enterohaemorrhagic *E. coli* O157:H7 and facilitates its adaptation to the host [42]. Another attaching-effacing pathogen, *C. rodentium*, also upregulates its virulence genes early during infection in a microbiota-dependent manner [43^••^]. In both *C. rodentium* and *Vibrio cholerae*-induced diarrhea, passage through the gut allows the pathogens to efficiently colonize subsequent hosts [44,45]. “Hyperinfectious” *V. cholerae* can also persist in aquatic reservoirs — a phenotype associated with significant changes in the bacterial transcriptome, including a repression of chemotactic factors and upregulation of carbon metabolism [46].

In addition, pathogens may acquire virulence, fitness and antimicrobial resistance genes from the gut community, as they evolve under the selective pressures from host immune defence, microbial competition or antibiotic use. Transfer of antibiotic resistance genes by conjugative transposons has long been shown to occur extensively among pathogens and commensals, within the gut reservoirs of both humans and farm animals [47]. The hospital-associated pathogen *Enterococcus faecalis* V583 can also evolve in the intestinal tract by disseminating fluoroquinolone resistance and fitness-enhancing bacteriophages [48^•^,49]. Using whole-genome sequencing and phylogenetics, He et al. recently demonstrated the rapid evolution of an epidemic *C. difficile* strain (ribotype 027), fuelled by antibiotic use and the transfer of mobile genetic elements with other intestinal bacteria [50,51]. In addition, Stecher and colleagues combined 16S rRNA gene and shotgun genome sequencing to show that during enterobacterial blooms in the inflamed gut, pathogenic *Samonella* and commensal *E. coli* can efficiently exchange fitness genes via conjugative plasmids [52]. The intestinal ecosystem represents a rich, dynamic reservoir for pathogens to intermingle and exchange genetic materials, especially during dysbiosis-induced bloom [53]. Therefore limiting dysbiosis, especially in the hospital setting, may have broad implications for the control of emerging infectious diseases.

### Enhanced disease persistence and host-to-host transmission

Dysbiosis can promote pathogen transmission by increasing the levels of shedding and prolonging the infectious period. In both murine models of *Salmonella* and *C. difficile*-induced disease, antibiotic disruption of the microbiota leads to a remarkably high bacterial load (10^8^-10^9^ CFU/g of faeces) [15,25,54]. This phenomenon, also known as the “supershedder” phenotype, allows pathogens to transmit very effectively through direct contact or environmental contamination. For example, *C. difficile* supershedders can spread infection by releasing millions of infectious spores, which persist in the environment for long periods of months or even years [25,55]. In a clinical study involving VRE-infected patients, those shedding high bacterial levels have also been shown to contaminate their hospital surroundings [56]. In addition, the clearance of many pathogens including *Salmonella*, *C. difficile* and *C. rodentium* depends on the presence of a healthy microbiota [25,36^•^,43^••^]. As such, intestinal dysbiosis can also promote the spread of pathogens by allowing them to establish persistent infection within the host.

## Future perspectives on microbiota restoration

**Figure 2:**
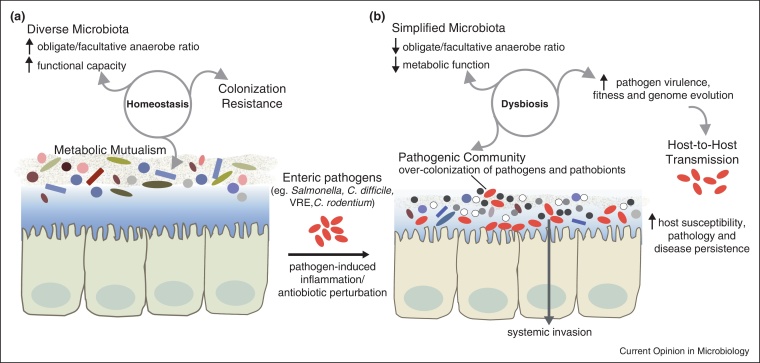
Features of intestinal dysbiosis during bacterial infections. **(a)** A healthy microbiota is typically diverse in structure and performs a wide range of functions (e.g. xenobiotic metabolism, production of SCFAs), thereby maintaining a mutualistic metabolic relationship with the host. Colonization resistance relies in part on the ability of the resident microbiota to outcompete pathogens for niches and nutrients. **(b)** During dysbiosis induced by pathogen-mediated inflammation or antibiotic perturbation, the microbiota is reduced in both taxonomic diversity and function, and intestinal colonization resistance is impaired. Diverse Gram-negative and Gram-positive pathogens can maintain dysbiosis by acting as keystone species to modulate community-wide shifts in the microbiota, possibly by orchestrating the host inflammatory response. As a result, the microbial community becomes more pathogenic, wherein pathogens and resident pathobionts may overgrow and even invade to cause systemic infection. Interactions with the gut microbiota often also allow pathogens to express their virulence factors and evolve under selective pressures. Consequently, the pathogens’ increased fitness and over-colonization may exacerbate pathology and enhance host-to-host transmission.

Each year, infectious diseases are increasingly difficult to treat because of rising antimicrobial resistance and a shortage in antibiotics discovery. Given the significant impact of dysbiosis on pathogen-mediated disease and transmission (Figure 2), the restitution of a healthy microbiota holds great promise as a therapy, at least for some infections. There are compelling evidences to suggest that administration of a diverse microbiota, or individual probiotic species, can restrict or eliminate enteric pathogens. For example, microbiota transplantation may be used in mice to prevent lethal disease caused by *C. rodentium* and VRE [57^•^,58^•^]. Further, patients with recurrent *C. difficile* infection, or murine supershedders, can completely eliminate *C. difficile* after receiving a healthy microbiota [25,59^•^]. Interestingly, *C. difficile* suppression may also be achieved by inoculating mice with Lachnospiraceae species or a defined group of six commensals (including the known probiotic *Lactobacillus reuteri* and previously uncharacterized Bacteroidetes and *Anaerostipes* spp.) [25,60]. This suggests that some microbial species derived from a healthy microbiota can potentially serve as standardized treatment, or “bacteriotherapy” [61]. The development of bacteriotherapy will require multiple complementary approaches, including high-throughput bacterial culturing (or “culturomics” [62^••^,63^•^]) and functional characterization of the human microbiota (e.g. pathway analyses of the microbiome, metatranscriptomics or metabolomics). Finally, mechanistic experiments in gnotobiotic animal models can inform us on how specific commensals influence colonization resistance and potentially be utilized as a microbiota-based therapeutic.

## References and recommended reading

Papers of particular interest, published within the period of review, have been highlighted as:

• of special interest
•• of outstanding interest

## Box 1 High-throughput methods to study host–microbiota interactions (Figure 1)

*16S rRNA gene sequencing*:

The 16S ribosomal RNA gene is highly conserved among prokaryotic species, and can be sequenced using various platforms (e.g. Roche 454 pyrosequencing, Illumina HiSeq/Miseq). By comparing to publicly available rRNA gene databases (e.g. the Ribosomal Database Project, SILVA, Greengenes), the 16S rRNA gene sequence can be used as a surrogate marker to define the microbial lineages present in a community. This allows researchers to analyse the microbiota structure, including its taxonomic and phylogenetic diversity. To classify 16S gene sequences into bacterial taxa (often called Operational Taxonomic Units — OTUs), one commonly assumes that those with ≥97% nucleotide identity can be assigned to a single species. This assumption largely holds true except for a few genera (e.g. *Bacillus*) in which distinct species only differ at a few bases of their 16S sequence. Another limitation is that organisms with significant polymorphism in the regions used for primer design, such as Actinobacteria and *Bifidobacteria* spp., are poorly detected with “universal” 16S gene primers [64].

*Shotgun sequencing*:

Bacterial DNA is broken into small fragments and sequenced. Fragment sequences are assembled into contigs and aligned to construct a complete genome. Traditionally, this method was used to study organisms isolated and grown in pure cultures. Therefore, genomic sequences were only obtained from cultured species and represent merely a snapshot of one bacterial clone rather than of the community as a whole. However, shotgun sequencing is being increasingly applied directly to DNA obtained from mixed-community samples (i.e. **metagenomics**). This technique allows us to understand the community structure and also to build pathways describing its function, especially when combined with mRNA or protein-based quantification of the microbiota. Thus metagenomics is gaining popularity as a method to study microbial ecosystems.

However, there are remaining challenges. Most environmental and biological communities exhibit vast diversity and unevenness, with large variations in the relative abundance of different members. Therefore the genomes of rare species, especially those with the potential to greatly influence its community or host (i.e. keystone species), may be poorly detected or assembled even with deep sequencing. Efforts are under way to improve assembly algorithms, detection and normalization of previously under-studied phylotypes. The current databases to classify microbial genes, enzymes and pathways (e.g. COG, KEGG, CAZy) are also rapidly expanding, which will enhance our ability to assign or predict the functional capacity of the microbiome from shotgun sequences.

*Culture-based methods*:

Despite the advent of culture-independent methods to study microbial communities, culture techniques still play an essential role in defining host–microbe interactions. Although only <30% of members of the human microbiota have been cultured to date, uncultured organisms may not be in fact unculturable. Permissive growth conditions for these organisms may be uncovered with improved, high-throughput culturing techniques [62^••^]. Microbial culturing can allow us to study biological aspects of the organism such as its growth, metabolism or behaviour in a given host. In addition, the whole genomes of these microbes may be sequenced to analyse for genetic traits (e.g. symbiosis or virulence genes), or evidence of genomic evolution.

Finally, the functional output of the microbiota may be assessed by sequencing the mRNA content (i.e. **metatranscriptomics**), quantifying the proteome (i.e. **metaproteomics**) or the active metabolites (i.e. **metabolomics**). These approaches are especially valuable for studying the microbiota function during human clinical trials.
